# Supplementary material for: Functional Characterization of Luciferase in a Brittle Star Indicates Parallel Evolution Influenced by Genomic Availability of Haloalkane Dehalogenase
Source: Mol Biol Evol. 2025 Apr 4;42(5):msaf081. doi: 10.1093/molbev/msaf081 (PMC12059646; doi:10.1093/molbev/msaf081)
Supplement: msaf081_Supplementary_Data [file msaf081_supplementary_data.pdf]

Supplemental Material for: ***Functional characterization of luciferase in a brittle star indicates parallel evolution influenced by genomic availability of haloalkane dehalogenase***

Emily S Lau[1*#], Marika Majerova[2,3#], Nicholai M Hensley[4], Arnab Mukherjee[5,6,7,8], Michal Vasina[2,3], Daniel Pluskal[2,3], Jiri Damborsky[2,3], Zbynek Prokop[2,3], Jérôme Delroisse[9,10], Wendy-Shirley Bayaert[9], Elise Parey[11], Paola Oliveri[11], Ferdinand Marlétaz[11], Martin Marek[2,3], Todd H Oakley[1*]

[*] Corresponding authors: Emily S Lau (emily.lau@lifesci.ucsb.edu), Todd H Oakley (oakley@ucsb.edu)

[#] These authors contributed equally: Emily S Lau and Marika Majerova

Affiliations

[1] Department of Ecology, Evolution, and Marine Biology, University of California Santa Barbara, Santa Barbara, CA 93106

[2] Loschmidt Laboratories, Department of Experimental Biology and RECETOX, Faculty of Science, Masaryk University, Kamenice 5, Bld. C13, 625 00 Brno, Czech Republic

[3] International Clinical Research Center, St. Anne's University Hospital Brno, Pekarska 53, 656 91 Brno, Czech Republic

[4] Department of Zoology, University of Cambridge, The Old Schools, Trinity Ln, Cambridge CB2 1TN, UK

[5] Department of Chemical Engineering, University of California Santa Barbara, Santa Barbara, CA 93106

[6] Department of Biological Engineering, University of California Santa Barbara, Santa Barbara, CA 93106

[7] Department of Chemistry, University of California Santa Barbara, Santa Barbara, CA 93106

[8] Neuroscience Research Institute, University of California Santa Barbara, Santa Barbara, CA 93106

[9] Biology of Marine Organisms and Biomimetics Unit, Biosciences Research Institute, University of Mons, Place du Parc 20, 7000 Mons, Belgium

[10] Laboratory of Cellular and Molecular Immunology, GIGA Research Institute, University of Liège, 4000 Liège, Belgium

[11] Centre for Life's Origins and Evolution, Department of Genetics, Evolution and Environment, University College London, London, UK

Supplemental Figures

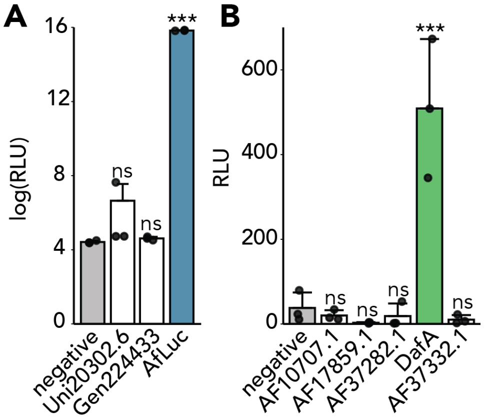

Supplemental Figure S1. Crude extracts of AfLuc and DafA exhibit significant luciferase activity with coelenterazine.

We recombinantly expressed dehalogenase genes from the genome of *Amphiura filiformis* and tested crude extracts for luciferase activity. (A) The negative control is a crude extract of bacteria transformed with an empty plasmid. (B) The negative control is a crude extract of bacteria expressing DhaA, a bacterial dehalogenase with no known luciferase activity. In both experiments, we tested for significant luciferase activity by using Dunnett's test (N = 3). Data in (A) are log-scaled. Data are expressed as average ± standard deviation. * denotes P < 0.05, ** denotes P < 0.01, *** denotes P < 0.001, and ns denotes non-significance (P ≥ 0.05).

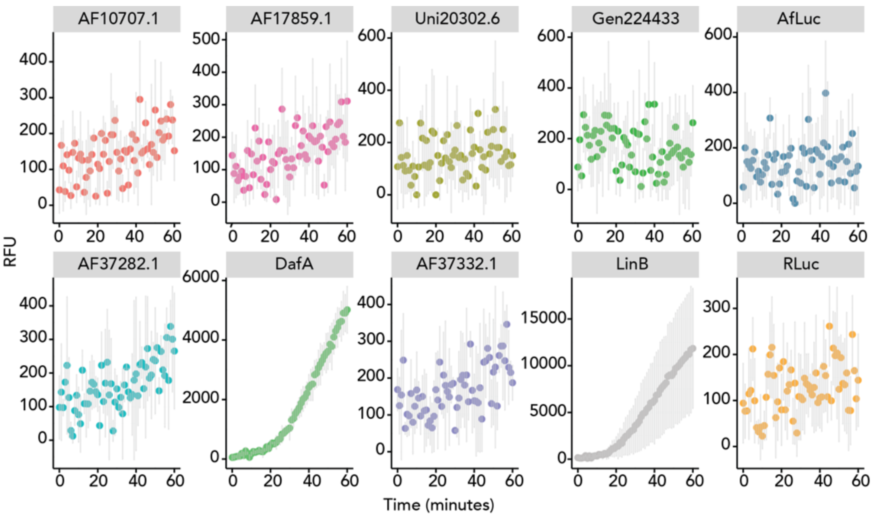

Supplemental Figure S2. DafA exhibits dehalogenase activity with the substrate 1,2-dibromoethane.

We recombinantly expressed each protein and tested crude cellular extracts at 30 ºC for dehalogenase activity using RLuc as a negative control and LinB, a bacterial dehalogenase, as a positive control. We monitored reactions for 60 minutes. Data are expressed as average ± standard deviation (N = 3).

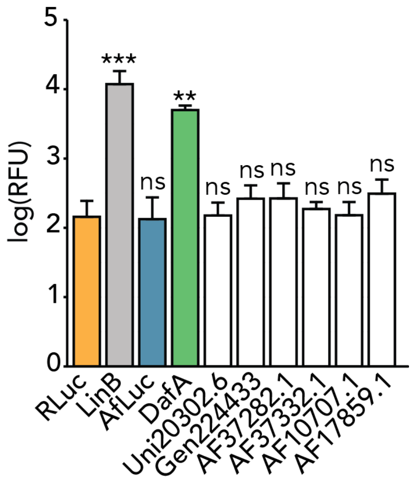

Supplemental Figure S3. Only DafA exhibits significant dehalogenase activity with the substrate 1,2-dibromoethane.

We compared the dehalogenase activity at 60 minutes for all recombinantly expressed dehalogenase sequences. Compared to the negative control of RLuc, only DafA significantly catalyzed the dehalogenation of 1,2-dibromoethane (Dunnett's test, $p = 0.00395$, $N = 3$). Data are log-scaled and expressed as average ± standard deviation. * denotes $P < 0.05$, ** denotes $P < 0.01$, *** denotes $P < 0.001$, and ns denotes non-significance ($P \geq 0.05$).

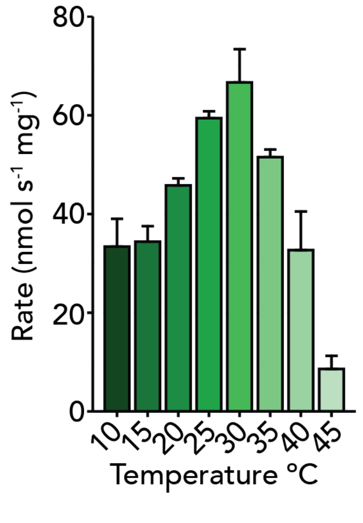

Supplemental Figure S4. DafA's dehalogenase activity varies across temperatures.

We measured DafA's dehalogenase activity with the substrate 1,2-dibromoethane across a range of temperatures. DafA exhibits maximum dehalogenase activity towards 1,2-dibromoethane at 30 ºC. The rate unit is nmol s^{-1} mg^{-1}. Data are expressed as means ± standard deviation ($N = 3$).

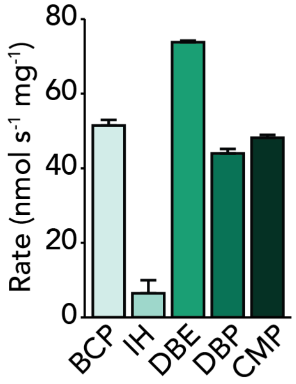

Supplemental Figure S5. DafA catalyzes the dehalogenation of multiple halogenated substrates

We tested DafA's ability to convert several halogenated substrates: (BCP = 1-bromo-3-chloropropane, IH = 1-iodohexane, DBE = 1,2-dibromoethane, DBP = 1,3-dibromopropane, CMP = 3-chloro-2-methylpropene). Rate is in nmol s^{-1} mg^{-1}. Data are expressed as the average ± standard deviation.

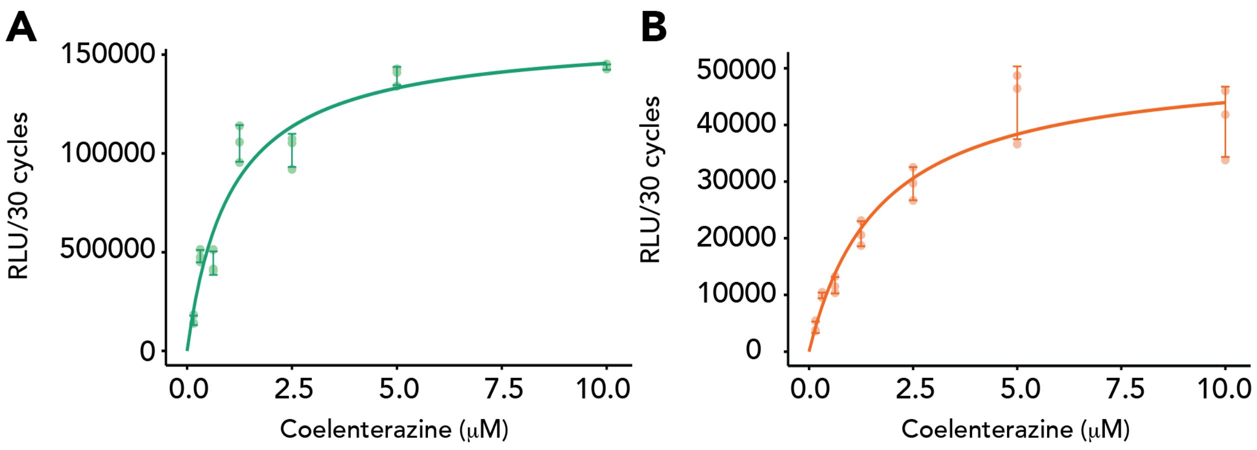

Supplemental Figure S6. Michaelis-Menten kinetic profiles for DafA (green) and PyroLuc (orange).

We varied the concentration of the coelenterazine substrate and measured light production for (A) DafA and (B) PyroLuc. For each measurement, we summed the light produced over 30 cycles (around 40 seconds).

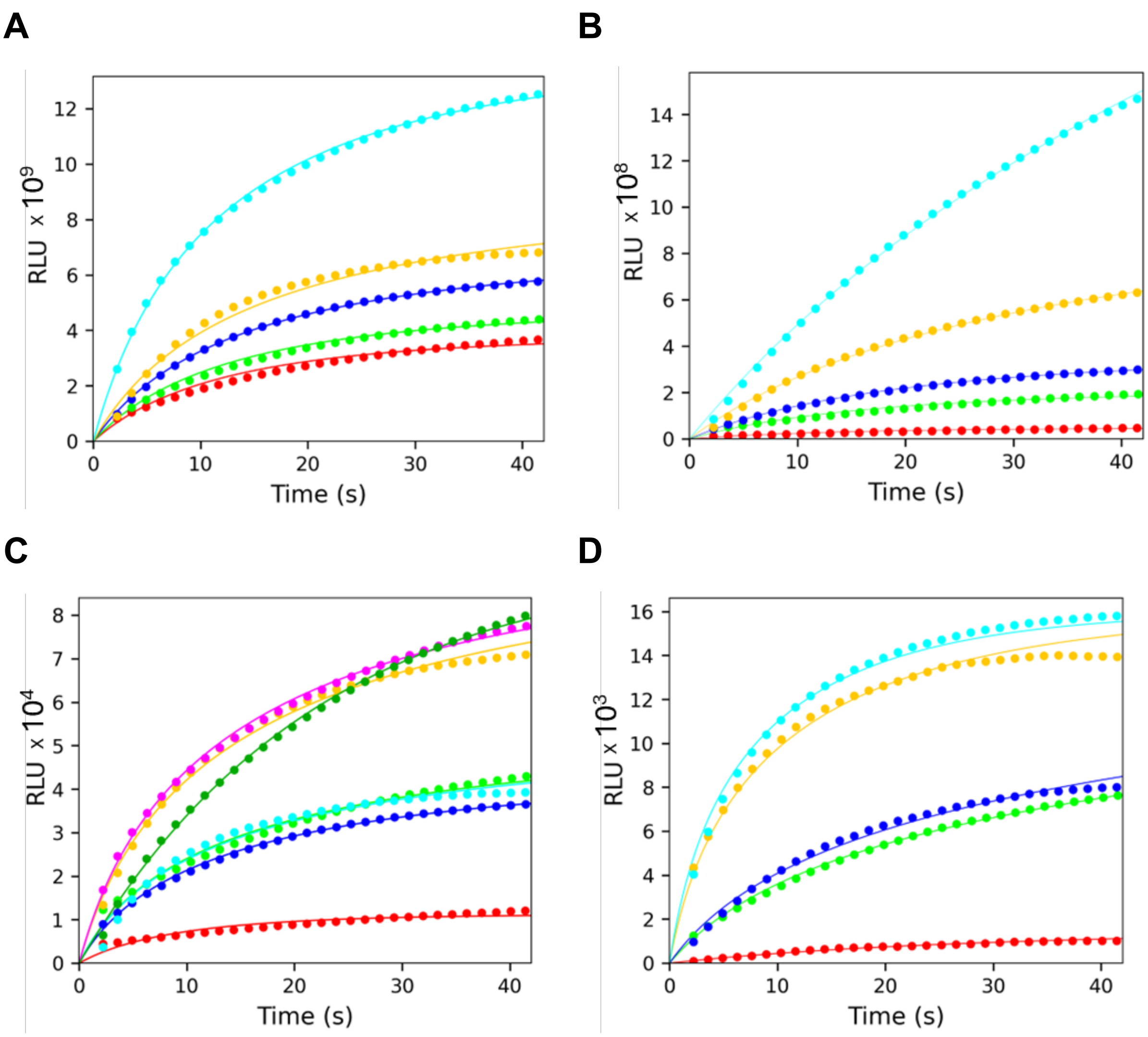

Supplemental Figure S7. Steady-state kinetic data.

Progress curves obtained by transformation of luminescence data recorded upon mixing 10 µM coelenterazine with 25 nM enzyme (final concentrations), (A) RLuc, (B) AfLuc, (C) DafA, and (D) PyroLuc. Each progress curve represents an average of 3 replicates. The solid lines represent the best fit.

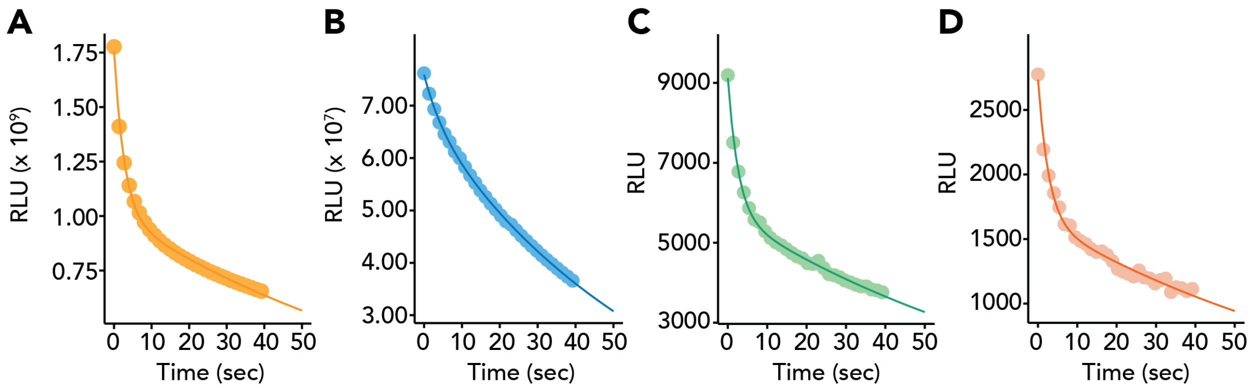

Supplemental Figure S8. Luminescence decay data fit to a biexponential decay model.

Results from estimating the luminescence decay parameters in (A) RLuc, (B) AfLuc, (C) DafA, and (D) PyroLuc. After combining 25 nM of recombinant protein with 10 μM coelenterazine, we recorded light production for around 40 seconds, averaged these data (N = 3), and fit them to a biexponential decay model.

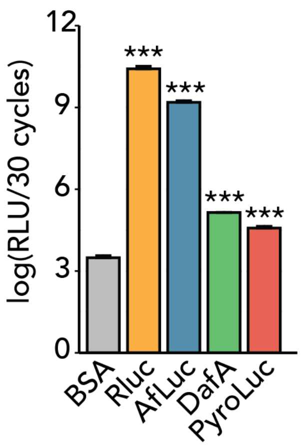

Supplemental Figure S9. RLuc, AfLuc, DafA, and PyroLuc exhibit significant luciferase activity.

Results of the luciferase assay show that RLuc (orange), AfLuc (blue), DafA (green), and PyroLuc (red) has significant luciferase activity with coelenterazine, relative to the negative control of BSA. We used Dunnett's test to assess significant luciferase activity (N = 3). Data are log scaled and expressed as means ± standard deviation. * denotes $P < 0.05$, ** denotes $P < 0.01$, *** denotes $P < 0.001$, and ns denotes non-significance ($P \geq 0.05$).

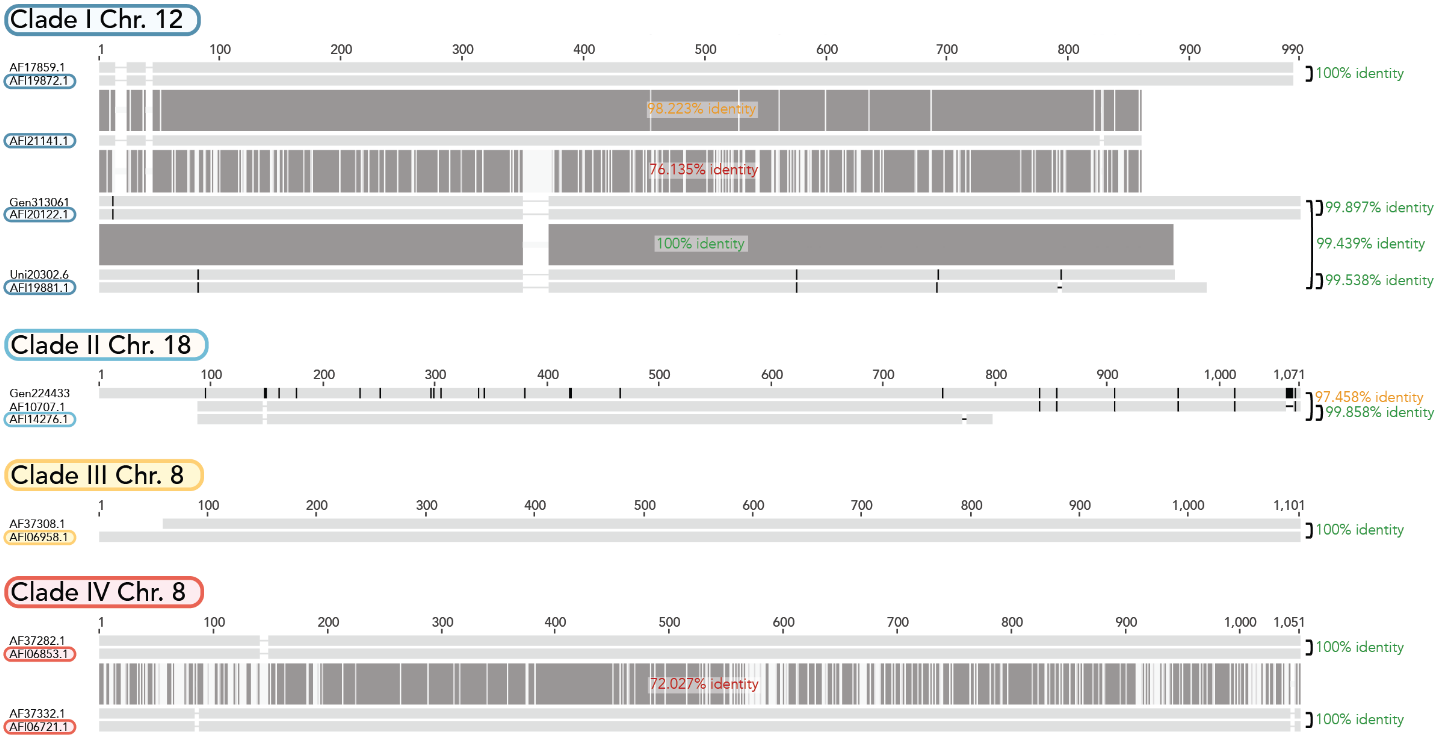

Supplemental Figure S10. Multiple sequence alignment of the coding regions in HLD/LUC genes from Parey et al. (2024) and previous sequences from an earlier genome assembly and transcriptome.

Gene models from the genome published in Parey et al. (2024) are highlighted and colored according to the chromosome in which they are found. Nucleotide differences between two synonymous sequences are denoted by black lines. Similarities between different pairs of gene models are denoted by gray lines. All HLD/LUC genes included in this figure are single-exon genes.

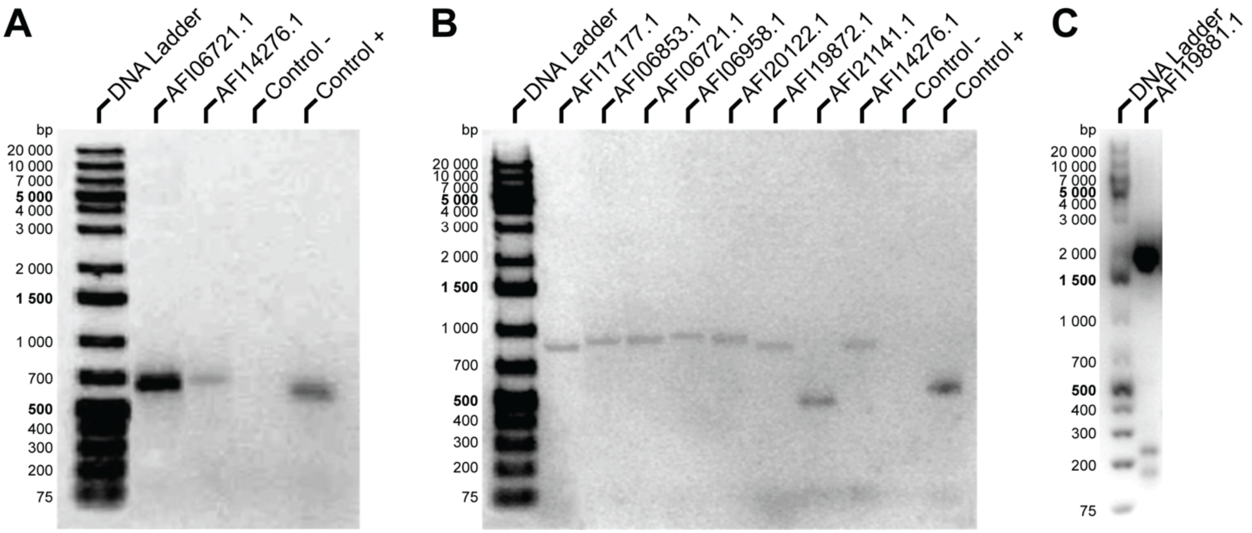

Supplemental Figure S11. Results from genomic DNA-based validation PCRs.

We performed PCRs to confirm portions of the HLD/LUC sequences. For all PCRs, we performed a negative control (absence of DNA) and positive control (amplification of a 600 bp fragment of the eukaryotic 18S ribosomal gene). All primer sets are detailed in Supplementary Table S6. (A) PCR amplification of AFI06721.1 and AFI14276.1 using primer set 1 produced single bands at the expected molecular weights. (B) PCR amplification of AFI17177.1, AFI06853.1, AFI06721.1, AFI06958.1 (*dafA*), AFI20122.1 (*afLuc*), AFI19872.1, AFI21141.1, and AFI14276.1 using primer set 2 produced single bands at the expected molecular weights. (C) PCR amplification of AFI19881.1 produced multiple bands — one at the expected molecular weight, and two other bands of lower molecular weight. However, these smaller bands did not impact downstream Sanger sequencing quality.

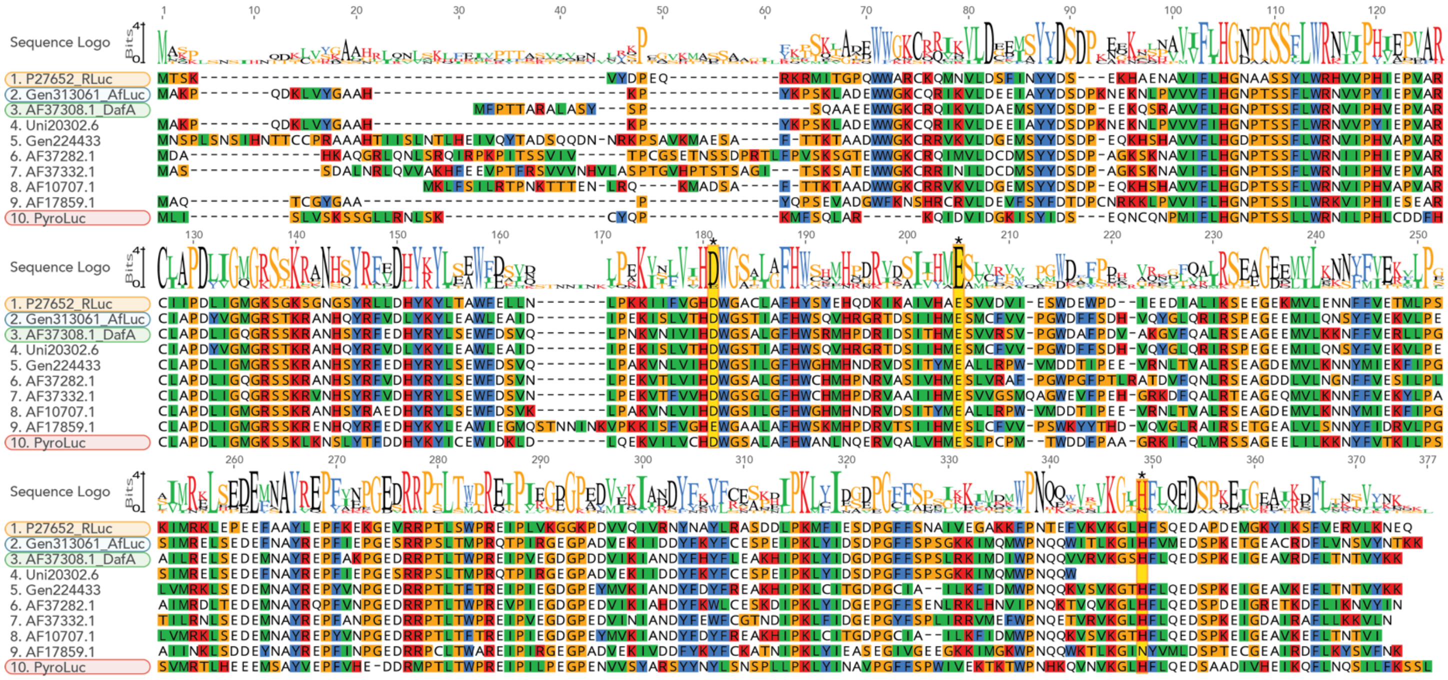

Supplemental Figure S12. Multiple sequence alignment of functionally tested proteins.

We used MAFFT to align *Renilla* luciferase (UniProt Accession P27652), PyroLuc, and protein sequences encoded by HLD/LUC genes in *Amphiura filiformis*. The catalytic triad of amino acids important for dehalogenase and luciferase activity are boxed in yellow and denoted with asterisks. The protein encoded by Uni20302.6 is a truncated protein, which is otherwise identical to the protein encoded by Gen313061/AfLuc and is missing a one of the three amino acids important for catalytic activity.

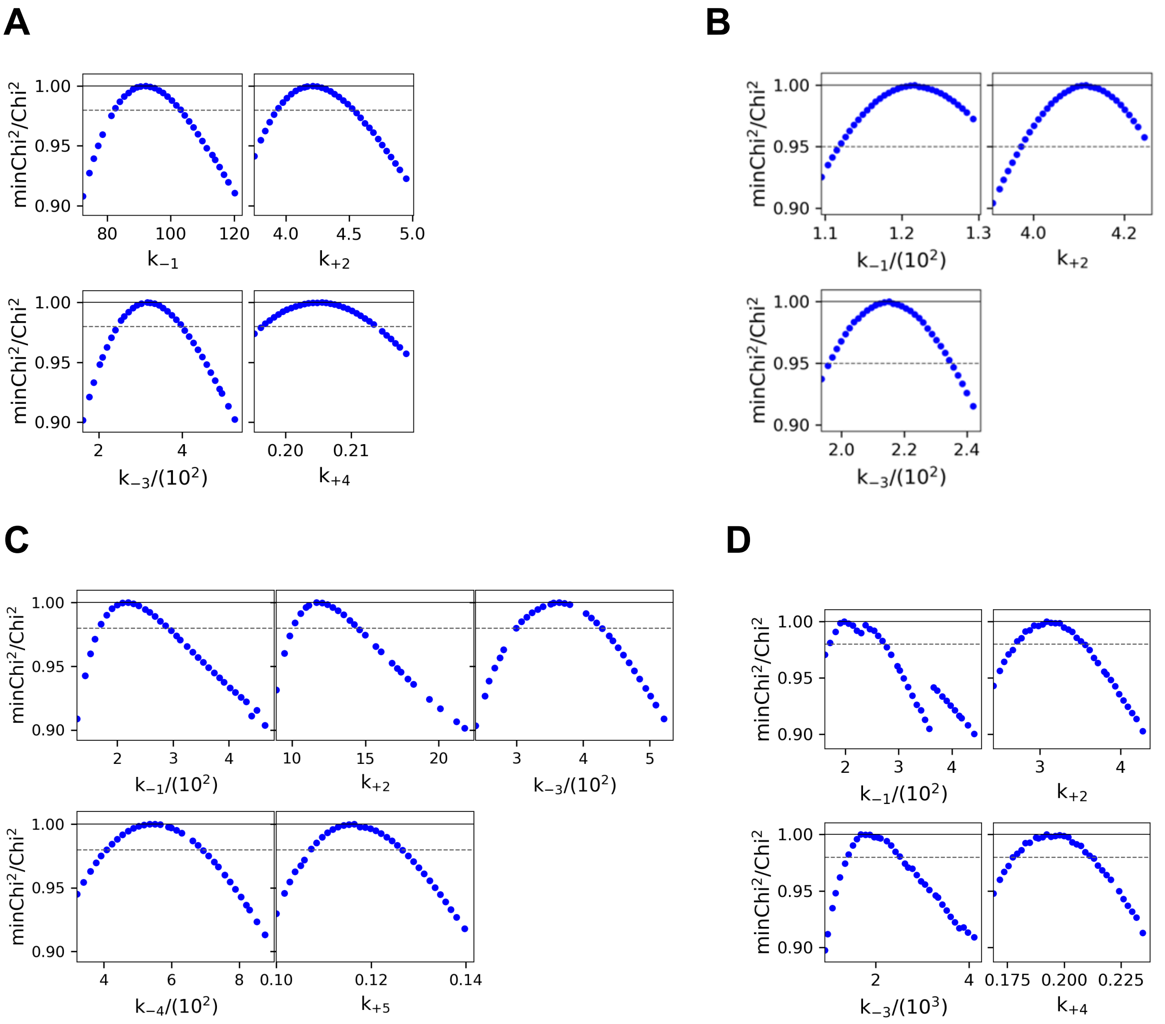

Supplemental Figure S13. Confidence contour analysis.

The confidence contours obtained for the steady-state parameters of (**A**) RLuc, (**B**) AfLuc, (**C**) DafA, and (**D**) PyroLuc. The gray dashed lines represent the χ^2 threshold of 0.98, defining the lower and upper limits for each parameter presented in Supplementary Table S4.

Supplemental Tables

Substrate specificities ($nmol.s^{-1}.mg^{-1}$)				
	DafA[1]	LinB[2]	DmmarA[3]	DnbA[4]
1-iodohexane	6.47 ± 3.50	22.02 ± 0.66	2.95 ± 0.07	1.05 ± 0.15
1,2-dibromoethane	73.79 ± 0.44	84.74 ± 2.82	1.94 ± 0.13	7.83 ± 0.30
1,3-dibromopropane	44.00 ± 1.19	63.26 ± 1.20	5.86 ± 0.00	6.48 ± 0.20
1-bromo-3-chloropropane	51.46 ± 1.51	52.95 ± 1.58	5.16 ± 0.11	4.77 ± 0.06
3-chloro-2-methylpropene	48.19 ± 0.78	43.34 ± 0.68	0.55 ± 0.17	1.35 ± 0.40

Supplemental Table S1. Specific dehalogenase activities of DafA enzyme and other well-characterized haloalkane dehalogenases towards several halogenated substrates.

DafA exhibits dehalogenase activity comparable to other characterized bacterial dehalogenases. The data are expressed as means ± the standard deviations.

[1] Data were measured at 30 ºC and obtained from this study

[2] Data were measured at 37 ºC and obtained in Buryska et al. (2019)

[3] Data were measured at 25 ºC and obtained in Vasina et al. (2022)

[4] Data were measured at 35 ºC and obtained in Vasina et al. (2022)

	RLuc	AfLuc	DafA	PyroLuc
K_M (μM)	0.91 ± 0.05	1.21 ± 0.03	2.0 ± 0.2	2.4 ± 0.3
lower - upper limit	0.83 - 1.03	1.12 -1.29	1.7 - 2.9	1.7 - 2.7
k_{cat} (s^{-1})	4.2 ± 0.2	4.12 ± 0.05	11 ± 1	3.0 ± 0.3
lower - upper limit	3.94 - 4.52	3.97 - 4.24	10.2 - 14.2	2.72 - 3.51
K_P (μM)	0.32 ± 0.04	0.47 ± 0.02	0.29 ± 0.06	0.05 ± 0.01
lower - upper limit	0.32 - 2.22	0.43 - 0.51	0.15 - 0.24	0.04 - 0.07
K_{SI} (μM)	n.d.	n.d.	5.9 ± 0.7	n.d.
lower - upper limit	n.d.	n.d.	4.2 - 6.8	n.d.
k_i (s^{-1})	0.21 ± 0.01	n.d.	0.12 ± 0.01	0.19 ± 0.01
lower - upper limit	0.20 - 0.21	n.d.	0.11 - 0.13	0.18 - 0.21

Supplemental Table S2. Steady-state kinetic parameters.

The estimated values ± standard errors calculated from the covariance matrix during nonlinear regression. The lower and upper limits for each parameter derived from the confidence contour at χ^2 threshold 0.98 (Supplemental Figure S13); n.d. = not determined.

	Lambda	Std error	p-value	Lambda_b	Std error	p-value	AICc
RLuc	1.160e-02	3.453e-03	0.00117	3.728e-01	1.301e-01	0.00524	3685.06
AfLuc	2.061e-01	2.940e-01	0.485	1.576e-02	3.411e-03	1.33e-05	3084.84
DafA	1.125e-02	3.317e-04	< 2e-16	3.800e-01	1.614e-02	< 2e-16	1092.20
PyroLuc	1.120e-02	2.269e-03	3.86e-06	3.703e-01	9.584e-02	0.00216	1211.83

Supplemental Table S3. Luminescence decay parameters estimated for RLuc, AfLuc, DafA, and PyroLuc.

We performed non-linear model fitting and tested four different non-linear models. Based on AICc and residuals, the bi-exponential decay model was the best fitting model for all the datasets.

Taxon		# of Origins of Coelenterazine Usage	Reference
Bacteria		0	
Fungi		0	
Radiolaria	Polycystine & Phaeodarian radiolarians	2	(Haddock et al. 2010)
Ctenophora		1	(Haddock et al. 2010)
Porifera		1	(Martini et al. 2020)
Octocorallia		1	(DeLeo et al. 2024)
Hexacorallia	*Parazoanthus*	1	(Cormier et al. 1973)
	Hormathiidae	1	(Bessho-Uehara et al. 2020)
Medusozoa	Hydrozoa	1	(Shimomura 2019)
	Periphylla periphylla (Scyphozoa)	1	(Shimomura et al. 2001)
	Pelagica noctiluca (Scyphozoa)	1	(Morin and Hastings 1971)
Chaetognatha	*Eukrohnia fowleri*	1	(Thuesen et al. 2010)
	Caecosagitta macrocephala	1	(Thuesen et al. 2010)
Mollusca	*Vampyroteuthis infernalis*	1	(Robison et al. 2003)
	Watasenia scintillans	1	(Tsuji 2002)
	Ommastrephidae	1	(Takahashi and Isobe 1994; Galeazzo et al. 2019)
	Pholas dactylus	1	(Tanaka et al. 2009)
Arthropoda	*Conchoecia pseudodiscophora* (Halocyprid ostracods)	1	(Oba et al. 2004)
	Copepod	1	(Oba et al. 2009)
	Oplophorus	1	(Shimomura et al. 1978)

	gracilirostris (Decapoda)		
	Gnathophausia ingens (Lophogastrida)	1	(Frank et al. 1984)
Echinodermata	*Amphiura filiformis* (Ophiuroidea)	1	(Delroisse et al. 2017)
	Thalassometra cf. *gracilis* (Crinoidea)	1	(Mallefet et al. 2023)
Chordata	*Oikopleura labradoriensis*	1	(Galt and Flood 1998)
	Etmopterus (Shark)	1	(Mizuno et al. 2021)
	Myctophidae (Myctophiformes)	1	(Duchatelet et al. 2019)
	Vinciguerria attenuata (Stomiiformes)	1	(Rees et al. 1990)

Supplemental Table S4. Number of origins of coelenterazine bioluminescence

Name of sequences tested in this manuscript	Most similar gene model In Parey et al. (2024)
"*afLuc*", the sequence Gen313061 in Delroisse et al. (2017)	AFI20122.1
"*dafA*", or preliminary gene model AF37308.1	AFI06958.1
AF17859.1	AFI19872.1
Uni20302.6 in Delroisse et al. (2017)	Ambiguous
AF10707.1	AFI14276.1
Gen224433 in Delroisse et al. (2017)	AFI14276.1
AF37282.1	AFI06853.1
AF37332.1	AFI06721.1

Supplemental Table S5. Synonymized sequences of preliminary gene models from a draft genome of *Amphiura filiformis* with the final gene models of the genome published in Parey et al. (2024).

We identified synonymous sequences based on percent sequence identity, as illustrated in Supplemental Figure S10.

Gene	Primers (5' → 3')	Product (nt)	PCR
AFI17177.1	*Forward* TTGGCACCAACTTCGAGCTC (set 2)<br>*Reverse* CCGATTTCAGCTGGGGAATCT (set 2)	884 (set 2)	1 band
AFI06853.1	*Forward* ACCTTTCACTTGCACCGTCT (set 2)<br>*Reverse* ACAAATCCGACCGAAGCCAA (set 2)	916 (set 2)	1 band
AFI06721.1	*Forward* GTCCATCCCACGTCAACCTC (set 1)<br>*Forward* AGTCCCTTCACTCTCACCGT (set 2)<br>*Reverse* TGCAAACGGTTCGCGATAAG (set 1)<br>*Reverse* GTCCCAACTTTCCGTAGCGT (set 2)	627 (set 1)<br>911 (set 2)	1 band
AFI06958.1 (*dafA*)	*Forward* AATCTCTGACAGCTTCCCCT (set 2)<br>*Reverse* GCACTCTGGCCGCATTAATG (set 2)	956 (set 2)	1 band
AFI20122.1 (*afLuc*)	*Forward* GCAAGCTTCTCCGGTCTCTT (set 2)<br>*Reverse* GGCAAAACCCCAGGACAAAC (set 2)	925 (set 2)	1 band
AFI19872.1	*Forward* AGCTTCTCCACACTCTGTTGG (set 2)<br>*Reverse* CTCTCATCGGTGTAGGGTGC (set 2)	859 (set 2)	1 band
AFI21141.1	*Forward* CTCTCATCGGTGTAGGGTGC (set 2)<br>*Reverse* TCAACACCAACGCTTCTCCA (set 2)	494 (set 2)	1 band
AFI19881.1	*Forward* AGAACGGTCGCCAAAAGAGT<br>*Reverse* AAGCGGTGAGTTGCAGTGTA	1604 (theoretically)	Multiple bands
AFI14276.1	*Forward* CATTCCTCATGTTGCGCCTG (set 1)<br>*Forward* TGACACTCACCTTCTGCTGG (set 2)<br>*Reverse* TGACACTCACCTTCTGCTGG (set 1)<br>*Reverse* TACTGCGGACTCCCAACAAG (set 2)	652 (set 1)<br>875 (set 2)	1 band

Supplemental Table S6. Primer sequences used to amplify HLD/LUC sequences from the genome of *Amphiura filiformis*.

Supplemental Materials and Methods

Materials

We purchased native coelenterazine (purity > 95 %, Catalog # 303-500) and NanoFuel Solvent (Catalog # 399) from Prolume LTD (Pinetop, AZ) and prepared single use aliquots by dissolving 500 µg of coelenterazine in 500 µL of nanofuel solvent to a stock concentration of 2.3615 mM, then stored the aliquots at -80 ºC. The pET21b(+)-Is-PETase was a gift from Gregg Beckham & Christopher Johnson (Addgene plasmid # 112202; http://n2t.net/addgene:112202 RRID:Addgene_112202). We purchased oligonucleotide primers and gBlocks™ from Integrated DNA Technologies (Coralville, IA). We purchased Q5 DNA polymerase (Catalog # M0491S), agarose gel extraction kit (Catalog # T1020S), reagents for Gibson assembly (Catalog # E2611L), competent *E. coli* 10-beta cells (Catalog # C3019H) for plasmid propagation, and competent *E. coli* BL21 cells (Catalog # C2527H) for protein expression from New England Biolabs (Ipswich, MA). We purchased reagents for extracting and purifying plasmids (Catalog # A1223) from Promega (Madison, WI) and reagents for Quick Start™ Bradford protein assay (Catalog # 5000205), the Bovine Serum Albumin (BSA) standard for calibration (Catalog # 500-0201), and Sodium dodecyl-sulfate polyacrylamide gel electrophoresis (SDS-PAGE) gels (Mini-PROTEAN TGX stain-free protein gels, Catalog # 4561094) from BioRad (Hercules, CA). We purchased Ni-NTA agarose (Catalog # R90115) and gravity-flow chromatography columns (Catalog # 34924) from Qiagen (Hilden, Germany). We purchased Amicon® Ultra centrifugal filter units with a 10 kDa molecular cutoff weight (Catalog # UFC901008) from Sigma Aldrich (St. Louis, MO).

Primer design, A. filiformis sampling, genomic DNA extraction and amplification of dehalogenase sequences

Based on the predicted HLD/LUC sequences of *A. filiformis*, several primer pairs were designed using the Primer3 software (v4.1.0, http://bioinfo.ut.ee/primer3). Primers were first *in silico* tested on the *A. filiformis* genome using the AmplifX software (v2.1.1 by Nicolas Jullien; Aix-Marseille Univ, CNRS, INP, Inst Neurophysiopathol, Marseille, France - https://inp.univ-amu.fr/en/amplifx-manage-test-and-design-your-primers-for-pcr). Primer pairs specifically amplifying a single target sequence were then selected. To confirm the sequences, validation PCRs were performed on genomic DNA of the brittle star. Due to its bacterial potential origin, these genes do not contain introns (Delroisse et al. 2017). Primer sequences are listed in Supplemental Table S6.

We collected *A. filiformis* (Müller, 1776) individuals with an Eckman grab at a depth of 30–40 m in the Gullmarsfjord near the Kristineberg Marine Research Station (University of Gothenburg, Fiskebäckskil, Sweden) in Summer 2023, rinsed sediments with fresh seawater to recover brittle stars, and maintained them in aquaria. We extracted genomic DNA from fresh brittle star arm fragments with the "DNeasy® Blood & Tissue" kit (QIAGEN), following the manufacturer protocol. We checked DNA integrity using gel electrophoresis, estimated

concentration using a microspectrophotometer (DS-11 DeNovix Spectrophotometer), and stored DNA at -20 °C. We amplified sequences of interest using the “Red'y'Star Mix” commercial kit (Eurogentecs), following the manufacturer protocol. For each PCR, we performed a negative control (DNA replaced by PCR water) and a positive control (primers targeting the eukaryotic 18S ribosomal RNA gene). The PCR cycle was as follows: 10 min at 95 °C, followed by 35 cycles, with each cycle consisting of 20 s at 94 °C, 30 s at the primer hybridization temperature (as determined using the AmplifX program), and 1 min at 72 °C; with a final extension step of 10 min at 72 °C. If no amplification was possible using the “Red'Y'Star Mix”, we performed PCR using using the Q5® High-Fidelity DNA Polymerase (New England BioLabs) and a touchdown PCR protocol to increase PCR sensitivity and yield. The PCR cycle was as follows: 4 min at 95 °C, followed by 22 cycles, with each consisting of 30 s at 95 °C, 30 s at the primer hybridization temperature + 11 °C – decreased temperature by 0.5 °C every 1 cycle, 1 min – 1 min 30 at 72 °C, followed by 13 cycles consisting each of 30 s at 95 °C, 30 s at the primer hybridization temperature, 1 min – 1 min 30 at 72 °C, with a final extension step of 7 min at 72 °C).

We loaded PCR products onto an 1% agarose Tris-Borate-EDTA gel and performed electrophoresis to assess the molecular weights of each obtained band. After electrophoresis, we stained the gel in a TBE solution containing a GelRed intercalating agent (1:3000 dilution; GelRed® Nucleic Acid Gel Stain, Biotium) for 30 minutes under agitation, and visualized the gel using the Bio Rad Gel Doc2000 Imaging system to identify fluorescent bands. For PCR products containing a single band, we purified them by using the “SmartPure PCR Kit” DNA Purification Kit (Eurogentecs). For PCR products containing multiple bands, we excised the band corresponding to the expected molecular weight, melted the agarose by incubating for 10 min at 80°C, and used the resulting mixture as a DNA template for a second PCR. We measured the concentration of purified DNA using microspectrophotometry and performed Sanger sequencing (Eurofins Genomics, Germany). Then, we aligned these sequences with the reference HLD/LUC genes of *A. filiformis* to verify their identity (using MAFFT G-INS-I, implemented in Geneious Prime v2024.0.5, 2005, Dotmatics, USA).

Cloning and expression of recombinant proteins

We codon-optimized and synthesized DNA sequences corresponding to the luciferase sequence of *Renilla reniformis* (UniProt Accession P27652) and dehalogenase sequences from *Amphiura filiformis*, namely Gen224433, Gen313061 (named *afLuc*), and Uni20302.6 as reported in (Delroisse et al. 2017), and AF10707.1, AF17859.1, AF37282.1, AF37308.1 (named *dafA*), AF37332.1 (protein sequences predicted from a draft genome of *A. filiformis*). We cloned these sequences into the bacterial expression vector pET21b, a plasmid that encodes a T7 promoter, ampicillin resistance, and a C-terminus hexahistidine tag for purification. We transformed competent *E. coli* NEB® 10-beta cells via electroporation to propagate plasmids, extracted and miniprepped the plasmids, and sequenced the plasmids (Genewiz, South Plainfield, NJ) to confirm successful cloning. Then, we transformed competent BL21 cells via electroporation with these plasmids for protein expression. We grew up transformed BL21 cells in Terrific Broth containing ampicillin (at a final concentration of 100 µg/mL) at 37 ºC (shaking at 250 rpm) to mid-log phage (optical density at 600 nm ~ 0.4), then added

isopropyl-β-D-thiogalactopyranoside (IPTG) to a final concentration of 1 mM to induce protein expression. We moved the bacterial cultures to a shaker at room temperature (250 rpm) and continued protein expression for 16-18 hours. We harvested bacterial cells by centrifuging cultures (4000 x g, 10 minutes) at 4 ºC, removed the supernatant, and froze the cell pellets. To lyse the bacterial cells, we resuspended the cell pellets in lysis buffer (10 mM TrisHCl, 500 mM NaCl, 0.5 % Tween20, 5 mM β-mercaptoethanol, pH 7.4) and sonicated the cells on ice (5 cycles of 30 s on, 10 s off at 30 % amplitude, MiSonix Sonicator 4000 with microtip probe). To pellet the cellular debris, we centrifuged the lysed cells (12,000 x g, 30 minutes) at 4 ºC, then collected the supernatant.

Testing crude extracts of recombinant proteins for luciferase activity

Testing Gen224433, Gen313061, and Uni20302.6

To test Gen224433, Gen313061, and Uni20302.6 for luciferase activity, we added 5 uL of 4 µM coelenterazine to 90 uL of the supernatant from lysed bacterial cells and measured luminescence using a microplate reader, set to an integration time of 3 seconds (Tecan Spark). As a negative control, we also tested an extract from BL21 cells transformed with an empty pQE80L plasmid. To test whether the expressed proteins exhibited luciferase assay, as compared to the negative control, we performed Dunnett's test (N = 3).

Testing AF10707.1, AF17859.1, AF37282.1, AF37308.1, and AF37332.1

To test AF10707.1, AF17859.1, AF37282.1, AF37308.1, and AF37332.1, we transferred single BL21(DE3) colonies of transformed cells into sterile 96-well plates (MTP) containing 100 µL of LB medium supplemented with ampicillin (100 µg/mL). We covered the plates with an air-pore membrane and cultivated for 3 h at 37 °C and 200 rpm. To induce the protein expression, we added 100 µl of LB medium with ampicillin (100 µg/mL) and IPTG (2 mM) to the mini-cultures and incubated MTP at 20 °C, 200 rpm, for 18 hours. We harvested cultures by centrifugation at 4 °C, 1600 g for 20 min, and froze the pellet immediately at -70 °C. After several hours we thawed the pellets at laboratory temperature for 10 min, and added 70 µL of a lysis buffer (20 mM potassium phosphate, 20 mM Na_2SO_4, and 1 mM EDTA, pH = 8.0) containing lysozyme (1 mg/mL) to each well. We incubated the MTP plate at 23 °C for 1 hour and removed cell debris from the lysate by centrifugation at 1600 g for 20 min. We transferred the supernatant into a new MTP and discarded the pellet. We used cell-free extracts from cells transformed with pet21b::DhaA and pet21b::RLuc as the negative and positive control, respectively. To test luciferase activity, we transferred 25 µL aliquots of cell-free extracts into a new MTP plate. We measured the baseline luminescence signal using FLUOstar Omega Microplate Reader (BMG labtech, Germany) for 5 s, with a gain set to 3200. Then, we added 225 µL of assay buffer (100 mM potassium phosphate, 1 mM Na_2SO_4, pH = 7.5) supplemented with 8.8 µM coelenterazine to the measured samples and immediately measured luminescence for 70 s. We performed all measurements in triplicates and then calculated the relative activity based on the peak area. We reported the results as averages along with their standard deviations. To test

whether the expressed proteins exhibited luciferase assay, as compared to the negative control, we performed Dunnett's test (N = 3).

Recombinant expression and purification of AfLuc, RLuc, DafA, and PyroLuc

We grew up BL21 cells, transformed with plasmids encoding RLuc, AfLuc, DafA, and PyroLuc, in 50 mL of Terrific Broth (containing ampicillin at a final concentration of 100 µg/mL) at 37 ºC (shaking at 250 rpm). To induce protein expression, we added 50 µL of IPTG to a final concentration of 1 mM. We moved the bacterial cultures to room temperature and continued shaking at 250 rpm for 18 hours. To harvest the bacterial cells, we centrifuged the cultures (4,000 x g, 10 minutes) at 4 ºC, removed the supernatant, and froze the cell pellets at -20 ºC for 3 hours. We extracted recombinant protein by lysing bacterial cells in 5 mL of lysis buffer (10 mM TrisHCl, 500 mM NaCl, 30 mM imidazole, 0.5 % Tween20, 5 mM β-mercaptoethanol, pH 7.4), then sonicated the cells on ice (5 cycles of 30 s on, 10 s off at 30 % amplitude, MiSonix Sonicator 4000 with microtip probe), and centrifuged the crude lysate (12,000 x g, 30 minutes) at 4 ºC to pellet cellular debris. To purify the proteins, we collected the clarified supernatants, added it to 1 mL of Ni-NTA agarose beads, then mixed and incubated the samples at 4 ºC overnight on a rotary mixer. Next, we loaded the Ni-NTA slurry into gravity-flow chromatography columns, allowed the slurry to settle, then removed the cap and discarded the flow through. We washed the column twice, each time with 2 mL of wash buffer (10 mM TrisHCl, 500 mM NaCl, 30 mM imidazole, pH 7.4), and eluted proteins bound to the Ni-NTA agarose by adding 4 mL of elution buffer (10 mM TrisHCl, 500 mM NaCl, 500 mM imidazole, pH 7.4). We performed spin ultrafiltration, using centrifugal ultrafiltration units with a 10 kDa molecular weight cutoff, to concentrate and buffer exchange the eluates into a storage buffer (10 mM TrisHCl, 300 mM NaCl, 20 % glycerol). We ran SDS-PAGE to assess protein purity and performed a Bradford assay (Bio-Rad Laboratories, Inc.), calibrated by using a standard curve of BSA, to quantify yield of recombinant proteins. We flash froze single-use aliquots of recombinant protein and stored them in - 80 ºC until use.

Collecting data for estimating Michaelis-Menten kinetics profiles

We characterized the enzyme kinetics of AfLuc, DafA, and PyroLuc, using RLuc as a positive control and BSA and luciferin as negative controls. We added 50 µL of recombinant protein to 50 µL of coelenterazine, resulting in a total volume of 100 µL and final concentrations of 25 nM recombinant protein in 1X TBS (pH 7.4) and varying concentrations of coelenterazine (10 µM, 5 µM, 2.5 µM, 1.25 µM, 0.625 µM, 0.3125 µM, 0.15625 µM). To perform the measurements, we used a plate reader (Tecan Spark) and first measured the background for 5 cycles, then injected coelenterazine and measured luminescence for 30 cycles, with a 1 second integration time for each cycle. We subtracted the background luminescence from each of the measurements after coelenterazine was injected. We repeated each sample measurement in triplicate.

Kinetic data analysis and statistics

An updated protocol applying new standards for collecting and fitting steady-state kinetic data (Johnson 2019; Schenkmayerova et al. 2021) was used to analyze and compare kinetic behavior of RLuc, AfLuc, PyroLuc and DafA. The recorded luminescence traces (rate vs. time) were transformed to reaction progress curves corresponding to cumulative luminescence in time (Supplemental Figure S7). The transformed kinetic data (product vs. time) were fitted globally with the KinTek Explorer (KinTek Corporation, USA). The software allows for the input of a given kinetic model via a simple text description, and the program then derives the differential equations needed for numerical integration automatically. Numerical integration of rate equations searching a set of kinetic parameters that produce a minimum χ^2 value was performed using the Bulirsch–Stoer algorithm with adaptive step size, and nonlinear regression to fit data was based on the Levenberg–Marquardt method (Johnson et al. 2009a). To account for fluctuations in experimental data, enzyme or substrate concentrations were slightly adjusted (± 10%) to derive best fits. Residuals were normalized by sigma value for each data point. The standard error (S.E.) was calculated from the covariance matrix during nonlinear regression. In addition to S.E. values, more rigorous analysis of the variation of the kinetic parameters was accomplished by confidence contour analysis by using FitSpace Explorer (KinTek Corporation, USA). In these analyses (Supplemental Figure S13), the lower and upper limits for each parameter were derived (Supplemental Table S2) from the confidence contour obtained from setting χ^2 threshold at 0.98 (Johnson et al. 2009b). The scaling factor, relating luminescence signal to product concentration, was applied as one of the fitted parameters, well constrained by end-point levels of kinetic traces recorded at particular substrate concentrations. The steady-state model (Supplementary Scheme S1) was used to obtain the values of turnover number k_{cat}, Michaelis constant K_M and equilibrium dissociation constant for enzyme-product complex K_p. A conservative estimate for diffusion-limited substrate and product binding k_1 and k_{-5} (100 $\mu M^{-1}.s^{-1}$) was used as a fixed value to mimic rapid equilibrium assumption. The basic kinetic model was extended by irreversible inactivation (significant for all enzymes except AfLuc) and substrate inhibition (indicated for DafA).

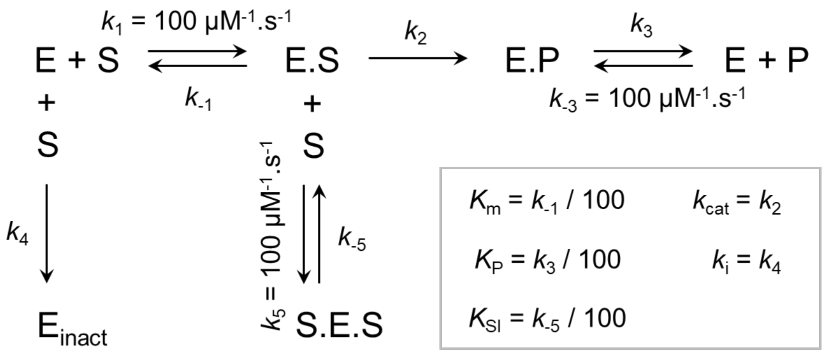

Supplementary Scheme S1. Steady-state model used for kinetic data analysis.

Measuring luminescence emission spectra

We measured emission spectra using a custom spectroradiometer set-up at UCSB, as detailed in a previous study (Hensley et al. 2021). In brief, we added 10 µL of 100 µM coelenterazine to 200 µL of purified recombinant proteins, diluted in 1X TBS pH 7.4 (final concentrations 4.76 µM coelenterazine with 1.17 µM Renilla luciferase, 0.68 µM AfLuc, 2.06 µM DafA, or 0.11 µM PyroLuc), and measured the emission spectra using a spectroradiometer (Acton SpectraPro 300i) with a charge-coupled device camera detector (Andor iDus). We corrected these spectral data using correction factors calculated from the spectrum of a black body-like light source (Ocean Optics LS-1) and subtracted background emission spectra data of 1X TBS buffer from the experimental data. We repeated each sample measurement in triplicate, then normalized and averaged these data.

Testing whole cell extracts for dehalogenase activity

To prepare whole cell extracts, we transferred transformed BL21(DE3) cells into sterile 96-well microtiter plates (MTPs) containing 100 µL of LB medium supplemented with ampicillin (100 µg/ml). We covered MTPs using an air-pore membrane and incubated them for 3 hours at 37 °C with shaking at 200 rpm. To induce protein expression, we added 100 µL of LB with ampicillin (100 µg/ml) and IPTG (2 mM) to each mini-culture and incubated MTPs at 20 °C for 18 hours, while shaking plates at 200 rpm. We harvested cell cultures by centrifuging MTPs (1,600 x g, 4 ºC, 20 minutes), and discarded the supernatant. To wash the cell pellets, we added 200 µL of reaction buffer (1 mM orthovanadate, 20 mM phosphate buffer, pH = 8.0), centrifuged MTPs (1,600 x g, 4 ºC, 20 minutes), and repeated this step again. To lyse cell pellets, we resuspended cell pellets in 200 µL of the reaction buffer and froze them at - 70 ºC.

To screen whole cell extracts for dehalogenase activity, we used a whole-cell halide oxidation (HOX) assay (Aslan-Üzel et al. 2020). We prepared the substrate 1,2-dibromoethane (DBE) in the reaction buffer (final concentrations 0.3 mM DBE, 1 mM orthovanadate, 20 mM phosphate buffer, pH = 8.0). In a new black bottom 96-well MTP, we added the assay master mix, which consists of 25 µM aminophenyl fluorescein, 26 mM H_2O_2, 1.1 U *Curvularia inaequalis* histidine-tagged vanadium chloroperoxidase, 1 mM orthovanadate, 20 mM phosphate buffer, pH = 8.0. We then added resuspended cells to each well for a final OD600 ~ 0.02, followed by 100 µL of DBE substrate to a final volume of 200 µL, and covered the plate. We immediately measured fluorescence with an excitation at 488 nm and emission detention at 525 nm, at 30 ºC (Synergy™ H4 Hybrid Microplate Reader), with results normalized to OD600 = 1. We measured all data in triplicate and calculated means and standard deviations.

Measurements of specific dehalogenase activity at varying temperatures

We measured the temperature profile and dehalogenase activity with various substrates for DafA using the capillary-based droplet microfluidic platform MicroPEX (Vasina et al. 2022), which enables us to measure specific enzyme activity within droplets for multiple enzyme variants in one run. We generated droplets (Mitos Dropix) and used a microfluidic pump to generate a custom sequence of droplets (150 nl aqueous phase, 300 nl oil spacing). These droplets are then incubated with the halogenated substrate via microdialysis and partitioning between the oil (FC 40) and the aqueous phase. This reaction solution consists of 1 mM HEPES, 20 mM Na_2SO_4, pH 8.2, and a complementary fluorescent indicator 8-hydroxypyrene-1,3,6-trisulfonic acid (50 µM HPTS). Then, fluorescence is measured using an optical setup with an excitation laser (450 nm), a dichroic mirror with a cut-off at 490 nm filtering the excitation light, and a Si-detector. Using this pH-based fluorescence assay, we were able to monitor enzymatic activity over four minutes. We processed raw data using LabView and used MatLab to calculate specific activities.

Supplemental References

Aslan-Üzel AS, Beier A, Kovář D, Cziegler C, Padhi SK, Schuiten ED, Dörr M, Böttcher D, Hollmann F, Rudroff F, et al. 2020. An Ultrasensitive Fluorescence Assay for the Detection of Halides and Enzymatic Dehalogenation. *ChemCatChem* 12:2032–2039.

Bessho-Uehara M, Francis WR, Haddock SHD. 2020. Biochemical characterization of diverse deep-sea anthozoan bioluminescence systems. *Mar. Biol.* 167:114.

Buryska T, Vasina M, Gielen F, Vanacek P, van Vliet L, Jezek J, Pilat Z, Zemanek P, Damborsky J, Hollfelder F, et al. 2019. Controlled Oil/Water Partitioning of Hydrophobic Substrates Extending the Bioanalytical Applications of Droplet-Based Microfluidics. *Anal. Chem.* 91:10008–10015.

Cormier MJ, Hori K, Karkhanis YD, Anderson JM, Wampler JE, Morin JG, Hastings JW. 1973. Evidence for similar biochemical requirements for bioluminescence among the coelenterates. *Journal Cellular Physiology* 81:291–297.

DeLeo DM, Bessho-Uehara M, Haddock SHD, McFadden CS, Quattrini AM. 2024. Evolution of bioluminescence in Anthozoa with emphasis on Octocorallia. *Proc. Biol. Sci.* 291:20232626.

Delroisse J, Ullrich-Lüter E, Blaue S, Ortega-Martinez O, Eeckhaut I, Flammang P, Mallefet J. 2017. A puzzling homology: a brittle star using a putative cnidarian-type luciferase for bioluminescence. *Open Biol.* [Internet] 7. Available from: http://dx.doi.org/10.1098/rsob.160300

Duchatelet L, Hermans C, Duhamel G, Cherel Y, Guinet C, Mallefet J. 2019. Détection de la coelentérazine chez cinq espèces de Myctophidae du plateau des Iles Kerguelen, Coelenterazine detection in five myctophid species from the Kerguelen Plateau. In: Second Kerguelen Plateau Symposium on Marine Ecosystems and Fisheries. November 2017, Hobart, Tasmania. archimer.ifremer.fr. Available from: https://archimer.ifremer.fr/doc/00502/61405/

Frank TM, Widder EA, Case JF. 1984. Dietary maintenance of bioluminescence in a deep-sea mysid. *J. Exp. Biol.* 109:385–389.

Galeazzo GA, Mirza JD, Dorr FA, Pinto E, Stevani CV, Lohrmann KB, Oliveira AG. 2019. Characterizing the Bioluminescence of the Humboldt Squid, Dosidicus gigas (d’Orbigny, 1835): One of the Largest Luminescent Animals in the World. *Photochem. Photobiol.* 95:1179–1185.

Galt CP, Flood PR. 1998. Bioluminescence in the Appendicularia. *The Biology of Pelagic Tunicates*:215–229.

Haddock SHD, Moline MA, Case JF. 2010. Bioluminescence in the Sea. *Ann. Rev. Mar. Sci.* 2:443–493.

Hensley NM, Ellis EA, Leung NY, Coupart J, Mikhailovsky A, Taketa DA, Tessler M, Gruber DF, De Tomaso AW, Mitani Y, et al. 2021. Selection, drift, and constraint in cypridinid luciferases and the diversification of bioluminescent signals in sea fireflies. *Mol. Ecol.* 30:1864–1879.

Johnson KA. 2019. New standards for collecting and fitting steady state kinetic data. *Beilstein J. Org. Chem.* 15:16–29.

Johnson KA, Simpson ZB, Blom T. 2009a. Global kinetic explorer: a new computer program for dynamic simulation and fitting of kinetic data. *Anal. Biochem.* 387:20–29.

Johnson KA, Simpson ZB, Blom T. 2009b. FitSpace explorer: an algorithm to evaluate multidimensional parameter space in fitting kinetic data. *Anal. Biochem.* 387:30–41.

Mallefet J, Martinez-Soares P, Eléaume M, O'Hara T, Duchatelet L. 2023. New insights on crinoid (Echinodermata; Crinoidea) bioluminescence. *Front. Mar. Sci.* [Internet] 10. Available from: https://www.frontiersin.org/journals/marine-science/articles/10.3389/fmars.2023.1136138/full

Martini S, Schultz DT, Lundsten L, Haddock SHD. 2020. Bioluminescence in an Undescribed Species of Carnivorous Sponge (Cladorhizidae) From the Deep Sea. *Frontiers in Marine Science* [Internet] 7. Available from: https://www.frontiersin.org/articles/10.3389/fmars.2020.576476

Mizuno G, Yano D, Paitio J, Endo H, Oba Y. 2021. Etmopterus lantern sharks use coelenterazine as the substrate for their luciferin-luciferase bioluminescence system. *Biochem. Biophys. Res. Commun.* 577:139–145.

Morin JG, Hastings JW. 1971. Biochemistry of the bioluminescence of colonial hydroids and other coelenterates. *J. Cell. Physiol.* 77:305–312.

Oba Y, Kato SI, Ojika M, Inouye S. 2009. Biosynthesis of coelenterazine in the deep-sea copepod, Metridia pacifica. *Biochem. Biophys. Res. Commun.* 390:684–688.

Oba Y, Tsuduki H, Kato S-I, Ojika M, Inouye S. 2004. Identification of the Luciferin--Luciferase System and Quantification of Coelenterazine by Mass Spectrometry in the Deep-Sea Luminous Ostracod Conchoecia pseudodiscophora. *Chembiochem* 5:1495–1499.

Parey E, Ortega-Martinez O, Delroisse J, Piovani L, Czarkwiani A, Dylus D, Arya S, Dupont S, Thorndyke M, Larsson T, et al. 2024. The brittle star genome illuminates the genetic basis of animal appendage regeneration. *Nature Ecology & Evolution*:1–17.

Rees JF, Thompson EM, Baguet F, Tsuji FI. 1990. Detection of Coelenterazine and Related Luciferase Activity in the Tissues of the luminous fish, Vinciguerria attenuata. *Comp. Biochem. Physiol.* 96A:425–430.

Robison BH, Reisenbichler KR, Hunt JC, Haddock SHD. 2003. Light production by the arm tips of the deep-sea cephalopod Vampyroteuthis infernalis. *Biol. Bull.* 205:102–109.

Schenkmayerova A, Pinto GP, Toul M, Marek M, Hernychova L, Planas-Iglesias J, Daniel Liskova V, Pluskal D, Vasina M, Emond S, et al. 2021. Engineering the protein dynamics of an ancestral luciferase. *Nat. Commun.* 12:3616.

Shimomura O. 2019. Bioluminescence: Chemical Principles and Methods. World Scientific

Shimomura O, Flood PR, Inouye S, Bryan B, Shimomura A. 2001. Isolation and properties of the luciferase stored in the ovary of the scyphozoan medusa Periphylla periphylla. *Biol. Bull.* 201:339–347.

Shimomura O, Masugi T, Johnson FH, Haneda Y. 1978. Properties and reaction mechanism of the bioluminescence system of the deep-sea shrimp Oplophorus gracilorostris. *Biochemistry* 17:994–998.

Takahashi H, Isobe M. 1994. Photoprotein of Luminous Squid, Symplectoteuthis oualaniensis and Reconstruction of the Luminous System. *Chem. Lett.* 23:843–846.

Tanaka E, Kuse M, Nishikawa T. 2009. Dehydrocoelenterazine is the organic substance constituting the prosthetic group of Pholasin. *Chembiochem* 10:2725–2729.

Thuesen EV, Goetz FE, Haddock SHD. 2010. Bioluminescent organs of two deep-sea arrow worms, Eukrohnia fowleri and Caecosagitta macrocephala, with further observations on bioluminescence in chaetognaths. *Biol. Bull.* 219:100–111.

Tsuji FI. 2002. Bioluminescence reaction catalyzed by membrane-bound luciferase in the “firefly squid,” Watasenia scintillans. *Biochimica et Biophysica Acta - Biomembranes* 1564:189–197.

Vasina M, Vanacek P, Hon J, Kovar D, Faldynova H, Kunka A, Buryska T, Badenhorst CPS, Mazurenko S, Bednar D, et al. 2022. Advanced database mining of efficient haloalkane dehalogenases by sequence and structure bioinformatics and microfluidics. *Chem Catalysis* 2:2704–2725.
